# Probiotic-Containing Nanofiber-Based Dental Floss Suppresses Subgingival Red Complex Periopathogens: A Randomized Double-Blind Crossover Trial

**DOI:** 10.1007/s12602-025-10898-4

**Published:** 2026-01-04

**Authors:** Filip Hromcik, Pavla Holochova, Jan Bohm, Eva Kuzelova Kostakova, Zdenek Pokorny, Filip Ruzicka, Petra Borilova Linhartova

**Affiliations:** 1 Clinic of Stomatology, Faculty of Medicine, St. Anne’s University Hospital, Masaryk University, Pekarska 53, Brno, 60200 Czech Republic; 2 Faculty of Science, RECETOX, Masaryk University, Kotlarska 2, 60200 Brno, Czech Republic; 3 Department of Chemistry, Faculty of Science, Humanities and Education, Technical University of Liberec, Studentska 2, Liberec, 46117 Czech Republic; 4 Department of Mechanical Engineering, Faculty of Military Technology, University of Defence, Kounicova 65, Brno, 61200 Czech Republic; 5 Institute of Microbiology, Faculty of Medicine, St. Anne’s University Hospital, Masaryk University, Pekarska 53, Brno, 60200 Czech Republic; 6 Clinic of Oral and Maxillofacial Surgery, University Hospital Brno, Masaryk University, Jihlavska 20, Brno, 62500 Czech Republic

**Keywords:** Probiotics, *Lactobacillus*, Nanotechnology, Dental devices, Oral health, Dysbiosis

## Abstract

Recently, a dental nanofloss containing a probiotic (*Ligilactobacillus salivarius*; *LS*-nanofloss) was developed by our team. This study aimed to evaluate: (i) its impact on subgingival plaque accumulation and gingival bleeding, and (ii) retention of selected subgingival periopathogens and the introduced probiotic bacteria, and (iii) its effect on the quantity of red complex periopathogens in the subgingival environment. This randomized, double-blind, crossover trial was conducted in 30 healthy male participants. Each subject underwent professional flossing and subsequently used *LS*-nanofloss/probiotic-free (control) nanofloss at two separate 14-day periods, always followed by a 14-day wash-out. Clinical assessments (subgingival plaque accumulation and gingival bleeding) were performed at five time points; subgingival plaque samples were collected at seven time points for quantitative analysis of total bacterial DNA, *L. salivarius*, a cariogenic bacterium *Streptococcus mutans*, and red complex periopathogens. Both nanofloss types significantly reduced plaque accumulation and gingival bleeding after 14 days (*p* < 0.05), especially in the anterior regions; these improvements, however, regressed after the wash-out period. *L. salivarius* DNA was detected in 93.3% of samples after professional flossing, 36.7% after self-flossing, and 13.3% even after the wash-out period. The use of *LS*-nanofloss was associated with a significant reduction in red complex periopathogens (*p* < 0.05). The investigated *L. salivarius*-containing nanofloss proved to be an effective oral hygiene device for subgingival introduction of this probiotic strain. Its application reduced the relative quantity of red complex periopathogens in the subgingival environment, showing potential to prevent the development of periodontitis. The trial was registered retrospectively on 2/9/2025 as NCT07149493.

**Supplementary Information:**

The online version contains supplementary material available at 10.1007/s12602-025-10898-4.

## Introduction

Individual dental hygiene remains the cornerstone of prevention and treatment of most oral (periodontal, dental, mucosal) diseases. Long-term plaque control supports gingival health and reduces the risk of caries development. At the same time, effective plaque management is essential in the treatment of periodontitis as well as in ensuring the longevity of restorative or prosthetic therapeutic efforts, since the development of both periodontitis and caries is linked to plaque accumulation and its unfavorable composition.

One strategy for countering oral dysbiosis, characterized by an increased presence of disease-associated pathogens [1–3], is to use oral probiotic bacteria. In dentistry, probiotics have been proposed as an adjunctive measure in the treatment of inflammatory periodontal disorders thanks to their ability to promote ecological shift from anaerobic gram-negative periopathogens, such as red complex bacteria *Porphyromonas gingivalis*, *Tannerella forsythia*, and *Treponema denticola* [4, 5], to gram-positive species [6, 7]. Probiotics have also been suggested for caries prevention and management due to their capacity to buffer pH and modulate dysbiotic biofilm composition by reducing cariogenic streptococci, especially *Streptococcus mutans* [8, 9].

Oral probiotic supplements are most often available as lozenges, chewing gums, toothpastes, or probiotic-containing mouthwashes and typically contain various strains of lactobacilli, streptococci, or bifidobacteria [10–12]. So far, *Ligilactobacillus salivarius* and *Limosilactobacillus reuteri* (formerly *Lactobacillus salivarius* and *Lactobacillus reuteri*, respectively) have been most thoroughly investigated with respect to the clinical efficacy in periodontology and cariology [13–19].

Direct application of probiotic bacteria into the gingival sulcus and interdental space was suggested to potentiate the beneficial impact of probiotics on biofilm composition. The tests of this approach yielded promising results [20, 21]. Sufficient retention of probiotic bacteria is required for the preservation of microbial eubiosis associated with positive immunomodulation in the oral environment, just like an adequate supply of bacteria for early recolonization of the tooth surface and sulcus after professional or self-performed oral hygiene [22]. A series of studies by Butera et al. reported improvement in subgingival microbial composition after non-targeted use of probiotic-containing toothpaste with chewing gum or mouthwash; however, a microbiological effect was only evident after 6 months of daily use [10, 11]. Targeted intrasulcular injection of probiotics has been also suggested as a possible strategy for introducing probiotic bacteria directly into the sulcus [20, 21, 23] with promising results. Still, a more user-friendly device available to both patients and dental professionals is needed to ensure good compliance and predictable clinical effect. Recently, a dental nanofloss containing a probiotic bacterium *L. salivarius* (*LS*-nanofloss) was developed by our team [24], using a probiotic strain embedded in the form of powder in a biocompatible and biodegradable nanofibrous shell of dental floss based on expanded nanofibers. Its effects related to oral health have, however, not been investigated so far.

To the best of our knowledge, no similar easy-to-use device for direct delivery of probiotics into the subgingival space is currently available. Butera et al. reported improvement in microbiological parameters in the subgingival space after the use of probiotic-containing toothpaste with chewing gum or mouthwash, the effect on red or orange complex bacteria was only significant after six months of use (after three months, the effect remained insignificant) [10, 11]. However, tools demonstrating more rapid effects are needed.

We hypothesized that 14-day use of *LS*-nanofloss, thanks to the direct delivery into the subgingival space, would lead to (i) reduced plaque accumulation and improvement of gingival health, (ii) enrichment of subgingival environment with the used probiotic strain presenting as an increase in the *L. salivarius* to red complex bacteria ratio and the increase in the relative quantity of *L. salivarius*, and (iii) decrease in the quantity of red complex bacteria in the subgingival environment. Thus, a protocol for testing the newly developed dental nanofloss, enriched or not enriched with a probiotic strain, was designed, aiming to: (a) clinically examine plaque presence and gingival bleeding in 30 participants at five time points (TPs) and (b) quantify the total content of bacterial DNA as well as DNA of five selected bacteria in subgingival plaque in seven TPs.

## Methods

This prospective randomized double-blind crossover study investigated the effect of 14-day use of the *L. salivarius*-containing nanofloss (*LS*-nanofloss) [24] and an identically produced probiotic-free nanofloss (control nanofloss) on selected parameters of oral health.

The study protocol and the informed consent were approved by the Ethics Committee of St. Anne’s University Hospital, Brno, Czech Republic (Approval No. 07–160222/EK, Project No. 14/22, approved February 16th, 2022), and the study was carried out in full accordance with the Helsinki Declaration of 1975, as revised in 2000. Informed consent was obtained from all participants.

### Nanofloss Specification

**Fig. 1:**
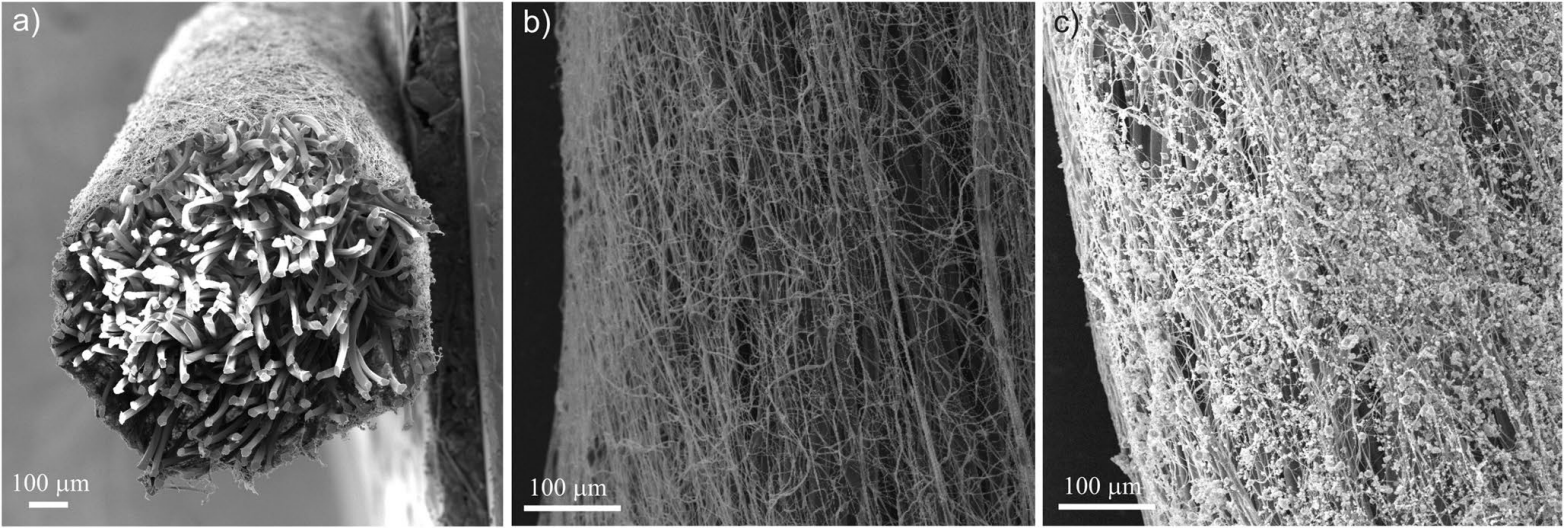
Scanning electron microscope images of the structure of the tested nanoflosses: (**a**) a cross-section of the composite thread – control nanofloss; (**b**) longitudinal view of the surface of the composite thread – control nanofloss; (**c**) longitudinal view of the surface of the composite thread with probiotics embedded on the surface of the nanofibrous shell – *LS-*nanofloss. Control nanofloss, probiotic-free nanofloss; *LS*-nanofloss, nanofloss with *Ligilactobacillus salivarius*

The nanofloss developed by our team is a composite yarn consisting of a core microfiber thread and a nanofiber shell. The core thread was a 1000D polyester expandable multifilament supplied by Richsource Biological Technology Co., Ltd (China). The nanofiber shell was deposited onto the core thread using alternating current (AC) electrospinning [25] from a 10 wt% solution of polycaprolactone supplied by Merck KGaA (Germany), molecular weight: 80,000 g/mol). The solvent system consisted of acetic acid, formic acid, and acetone in a 1:1:1 weight ratio. Polycaprolactone is a biocompatible and biodegradable aliphatic polyester. The composite thread (see Fig. 1a, b) was produced by AC electrospinning at a speed of 50 m/min. The distance between the spinning electrode and horizontally driven microfibre thread was 300 mm. Both twirling devices were operated at a rotational speed of 5000 rpm, while the yarn stress was maintained at 40 cN. The AC power supply operated at 50 Hz at an effective voltage of up to 40 kV. The ambient conditions during AC electrospinning were a temperature of 22 °C and relative humidity of 34% RH. The average nanofiber diameter was 380 ± 150 nm. Finally, the composite yarn was passed through a glass U-tube containing probiotic *L. salivarius-*based powder to produce *LS*-nanofloss (see Fig. 1c).

The fineness of the core microfiber thread without nanofibers and probiotics was 111 tex (1 m of thread weighed 111 mg). During AC electrospinning, 15 mg of nanofiber was deposited per meter of the core thread, and 7 mg of the probiotic powder was subsequently applied per meter of the composite yarn. For the evaluation study, both control nanofloss and *LS*-nanofloss were wound onto plastic spools and placed in 3D-printed boxes. The total length wound on each spool was 10 m.

### Recruitment, Inclusion, and Exclusion Criteria

The study aimed for a population of 30 participants recruited from the public and examined by a single periodontologist. The inclusion criteria were defined as follows: only Czech or Slovak adult men (≥ 18 years), at least 20 own teeth, periodontally healthy or mild periodontitis (maximum Stage 2 according to 2017 Classification) [26], good oral hygiene habits (brushing once or twice daily). Exclusion criteria were immunocompromised status (HIV positive, autoimmune disorders), diabetes mellitus, cardiovascular or oncologic disease, systemic and/or local oral antibiotic treatment within two months prior to the study. Only men were included in the study to prevent any misinterpretation of results associated with fluctuation of gingival bleeding during menstrual cycle [27].

All volunteers who met the criteria were thoroughly instructed on the study protocol and signed the informed consent. Throughout the study, the participants were instructed to refrain from mutual intimate oral contact, smoking, consuming raw onion or garlic, fermented food, and probiotics. They were also asked to abstain from alcohol 24 h before examination, and to avoid elective dental care during the study period.

A survey primarily identified 109 volunteers. As the study aimed for the analysis of samples from 30 participants and an approx. 10% dropout was expected, 33 individuals (divided into groups of 16 and 17 participants) meeting all inclusion and exclusion criteria and were included in the clinical part of the study. From each group, 15 participants were eventually randomly selected for the final analyses.

### Study Design

The participants were randomly assigned to Group I and Group II. Randomization was performed after inclusion into the study by the study nurse by drawing lots (balls from an opaque bag, 17 white – Group I – and 17 black – Group II – balls were present in the bag). The nanofloss packaging was labelled Group I or Group II, otherwise the packaging was identical. The threads were visually indistinguishable from each other. Both the periodontologist and the participants were fully blinded to the type of nanofloss throughout the whole study, the study nurse remained the only person to know the allocation of the patient. Laboratory staff and statisticians knew the Group I and Group II allocation but were blinded to the meaning of the designation.

**Fig. 2:**
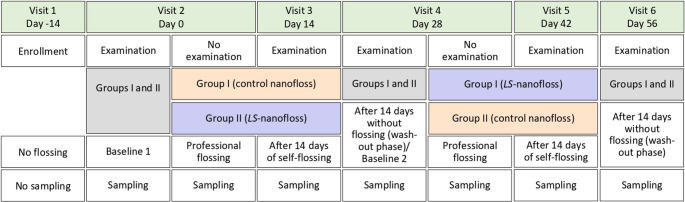
Study design with the timeline. Control nanofloss, probiotic-free nanofloss; *LS*-nanofloss, nanofloss with *Ligilactobacillus salivarius*

While Group I used the control nanofloss for the first two weeks and later (after a 14-day wash-out phase) the *LS*-nanofloss, this sequence was reversed in Group II, see Fig. 2. A 14-day wash-out period was included based on similarly designed studies [28] with respect to gingivitis onset, which requires 10–14 days to occur if patient refrains from any kind of oral hygiene [29].

The clinical assessment and sample collection were performed during six visits to the periodontal clinic (Fig. 2). Upon inclusion in the study (Day − 14, first visit), the participants were instructed to refrain from dental flossing and interdental brushing for 14 days. On the second visit (Day 0), samples of the subgingival plaque were collected, followed by a clinical examination and professional flossing (with a group-specific floss) by the periodontologist. Immediately after, subgingival plaque was sampled again, and the patients were instructed to keep using the assigned floss for 14 days. On the third visit (Day 14), plaque sampling and clinical evaluation were performed again, which was followed by a 14-day wash-out phase without flossing and interdental brushing. On the fourth visit (Day 28), identical procedures as during the second visit were performed using the other floss for professional flossing. The next stages (self-flossing with the other floss, the fifth visit with sample collection on Day 42, a second wash-out phase, and the last visit on Day 56) followed the same pattern as the first half of the study.

### Dental Hygiene Measures

During the study, all participants were required to adhere to standardized dental hygiene measures, i.e., brushing 2x daily with a soft-bristled manual toothbrush (CS Super Soft 3960, Curaden, Kriens, Switzerland) and a uniform toothpaste (elmex^®^ Caries Protection, Colgate-Palmolive, New York, US). No other additional devices (with the exception of the devices that were subject of the study), such as interdental brushes, flosses, toothpicks, oral irrigators, mouthwashes, etc., were allowed. During the wash-out periods, only manual toothbrushing with the specified toothbrush and toothpaste was permitted. Within the testing periods, additional use of an assigned floss was introduced.

Before the commencement of the study, all participants received professional instruction on proper brushing with the Bass technique [30] and on the correct flossing technique. They were asked to perform specified dental hygiene thoroughly twice daily.

### Clinical Examination and Sample Collection

Clinical parameters were recorded at five TPs (see Fig. 2) to assess the effects of flossing on plaque accumulation and gingival bleeding upon irritation. The modified Approximal Plaque Index (API) [31] and a modified Sulcus Bleeding Index (SBI) [32] were used for this purpose.

Subgingival dental plaque samples were collected at seven TPs (see Fig. 2). All samples were harvested using endodontic paper points (Sure-endo Absorbent Paper Points ISO 40, Sure Dent, Seongnam, South Korea) from the mesial aspects of the upper left first molars (tooth 26, FDI notation), ensuring that at least two-thirds of each paper point was covered with biological material. In one patient (ID TA01) in whom the tooth 26 was missing, the adjacent upper left second molar (tooth 27, FDI notation) was sampled instead. Each paper point was placed into a separate sterile vial and frozen at − 80 °C within 60 min of collection. The tooth 26 (upper left first molar) was selected as a worst-case scenario for cleaning as it is difficult to effectively reach with a floss (teeth in the posterior region—especially molars, and their interproximal spaces—are the sites most commonly affected by periodontitis). Therefore, it could be reasonably assumed that any introduction of the probiotic strain into the subgingival space of this tooth would be comparable or more effective in other teeth.

### DNA Extraction

DNA from 210 samples of 30 participants was extracted manually by the QIAamp DNA Mini Kit (QIAGEN, Hilden, Germany) according to the manufacturer’s instructions, with the addition of lysozyme (20 mg/mL) pre-treatment. The elution volume was 50 µL. Negative controls (NCs, containing DNA-free H_2_O, *N* = 20) and collection material controls (MCs, unused endodontic paper points, *N* = 10) underwent the same process.

The purity and concentration of the extracted DNA were determined using a spectrophotometer NanoDropND-1000 (Thermo Fisher Scientific, USA), and the DNA quality was assessed using gel electrophoresis. The extracted DNA was stored at − 20 °C.

### Multiplex qPCR Analysis

Multiplex qPCR assay targeting red complex bacteria (*P. gingivalis*, *T. forsythia*, and *T. denticola*) and total content of bacterial DNA is well-established in our department and details of the procedure were published previously [33]. Moreover, we modified another multiplex qPCR assay routinely performed in our laboratories serving for the detection and quantification of *S. mutans* [33]. The assay was modified by including *L. salivarius* – the probiotic species included in the *LS*-nanofloss – in the analysis. The bacterial strain used in the production of the *LS*-nanofloss was obtained from MicroCen Trans s.r.o. (Brno, Czech Republic) and was used as a template for the design of the *de novo* developed qPCR. Genomic DNA of *L. salivarius* strain L33 was extracted from cultured bacterial cells using the phenol-chloroform extraction with lysozyme (20 mg/mL) pre-treatment. The whole genome sequencing (WGS) of bacterial DNA was performed on a MiSeq instrument (Illumina, CA USA) using the Illumina DNA Prep for library preparation. The library was prepared according to the manufacturer’s instructions. The DNA input was 1 ng of bacterial DNA, Nextera DNA CD Indexes were used. The *L. salivarius* strain BCRC 14,759 (GCF_002736025.1, NCBI database record https://www.ncbi.nlm.nih.gov/datasets/genome/GCF_002736025.1/), with 99.8% of reads mapped, served as a standard for the newly developed multiplex qPCR panel.

For the newly developed assay, the oligonucleotides complementary to specific 16–23 S regions of individual bacteria were selected based on a previously published study [34]. The probe was designed *de novo* using the Primer-Blast online tool [35]. The sequences of the primers are shown in Supplementary Table S1. The amplified fragments from target regions of all bacteria were prepared by end-point PCR and cloned into the cloning vector pGEM^®^-TEasy (Promega, Madison, WI, USA). The correctness of these cloned DNA fragments was verified by sequencing using T7FW and M13R primers (KRD, Prague, Czech Republic). To determine the amplification efficiency and sensitivity of the assay, a ten-fold dilution series of plasmid DNA from all reference species was prepared, ranging from 5 × 10^8^ to 5 × 10^1^ copies/µL. The amplification efficiency ranged from 94% to 110%, with R^2^ calibration fit values > 0.990.

The multiplex qPCR analyses were carried out in a total volume of 20 µL consisting of 5 µL QuantiNova Multiplex PCR Kit (QIAGEN, Hilden, Germany), 0.25 µM primers for each bacterium except for 0.4 µM forward primer for *L. salivarius*, 0.2 µM of each probe, and 2 µL of the target genomic DNA extracted from samples and NCs. The primers and double-quenched TaqMan probes with reporter dyes were obtained from IDT (Integrated DNA Technologies, Inc., Coralville, IA, USA). The final qPCR conditions were as follows: 2 min at 95 °C, 40 cycles at 95 °C for 5 s, and 60 °C for 30 s with signal collection. All amplifications and detections were carried out on the LightCycler^®^ 480 Instrument II (Roche Diagnostics AG, Rotkreuz, Switzerland) using 96-well white reaction plates with LightCycler^®^ 480 Sealing Foil (Roche Diagnostics AG, Rotkreuz, Switzerland). Data were analyzed in the LightCycler^®^ 480 software v1.5. Each analysis was performed in duplicate, the variation in C_q_ for technical replicates was below 0.5 C_q_ cycle, which is in line with common practice [36]. Values below the detection threshold (< 50 copies/µL) were considered undetectable and replaced with zeros.

### Bioinformatic Analysis

WGS data were obtained in FASTQ format from sequencing of DNA extracted from the used bacterial strain of *L. salivarius*. For strain identification, raw sequencing data were processed as follows: the initial quality control of the raw sequencing reads was performed in FastQC (version 0.11.5), and the results were aggregated and visualized using MultiQC (version 1.8). Key quality metrics, such as per-base sequence quality, GC content, and sequence duplication levels, were assessed to ensure the raw data integrity. Adaptor sequences and low-quality bases were trimmed, and overall read quality was improved using FastP (version 0.23.2). Subsequently, FastP was used to filter out reads with a Phred score below 20 and to remove adaptors, ensuring that only high-quality, paired-end reads were retained for further analysis. High-quality reads were mapped against all complete genomes accessible through NCBI datasets under taxonomy ID 1624 to determine the bacterial strain. The alignment was performed in Bowtie2 (version 2.5.1). Sorting and indexing of the resulting BAM files were done in SAMtools (version 1.19.1). The analysis included a detailed comparison of the mapping results to identify the bacterial strain. The mapping quality metrics were compiled and compared across the different reference genomes. The strain was identified based on the reference genome with the highest quality metrics, including the number of mapped reads.

### Statistical Analysis

All statistical analyses were performed using the R programming language (version 4.1.2) [37] using the packages *lme4* (version 1.1.38), *lmerTest* (version 3.1.3), *ggplot2* (version 4.0.1), *patchwork* (version 1.3.1), *ComplexHeatmap* (version 2.18.0). *dplyr* (version 1.1.4) and *tidyr* (version 1.3.1). For all secondary and exploratory analyses, *p*-values were adjusted within each sub-analysis using the Benjamini–Hochberg method. Adjusted *p*-values of less than 0.05 were considered statistically significant. All tests were two-sided unless stated otherwise.

The limit of detection (LoD) for the qPCR assays was 50 copies/µL. Values below the LoD were treated as left-censored and imputed as LoD/2 (25 copies/µL). Relative quantities of individual taxa within a sample were obtained by dividing taxon-specific concentration by the total bacterial DNA concentration. Relative quantity of the red complex bacteria was defined as the combined relative quantity of *P. gingivalis*, *T. forsythia*, and *T. denticola*.

The primary microbiological endpoint was the within-subject treatment contrast in log-ratio change (post-treatment – baseline) of *L. salivarius* to red complex bacteria ratio after 14 days of nanofloss use. To account for period, sequence, and potential carry-over effects, log-ratio changes were analyzed using linear mixed-effects models with treatment (*LS*-nanofloss or control nanofloss), period (1 or 2), and sequence (*LS*-nanofloss→control nanofloss or control nanofloss→*LS*-nanofloss) as fixed effects and subject as a random intercept. Potential carry-over effects were evaluated by comparing treatment groups after first wash-out phase using Wilcoxon test. Estimated treatment effects are reported along with 95% Wald confidence intervals (CI). The relative quantities of *L. salivarius* and the red complex bacteria were assessed in the same way.

API and SBI were calculated for each patient, time point and tooth group (anterior vs. posterior). For each patient × time point × tooth group combination, the numbers of affected and unaffected teeth were used as binomial outcomes. To respect the binomial nature of these measurements and the 2 × 2 crossover structure, generalized linear mixed-effects models (GLMMs) with a binomial distribution and logit link were fitted. Odds ratios with 95% CI are reported.

Associations between clinical indices and microbiological measures were assessed using Spearman’s rank correlation across all patients and applicable TPs. For descriptive visualization, violin boxplots were used to display the distributions of relative quantity of the red complex bacteria, *L. salivarius*, their ratio, and API and SBI values at individual TPs or within each treatment period. Paired Wilcoxon signed-rank tests were used in these exploratory plots to annotate within-subject differences, and significance levels were indicated. These visualizations were intended to complement, but not replace, the formal mixed-model analyses described above.

## Results

### Descriptive Characteristics of Study Participants

At the baseline (TP1), Groups I and II did not differ in mean age (Group I: 32 ± 6 vs. Group II: 29 ± 8), BMI (Group I: 26.8 ± 3.7 vs. Group II: 25.1 ± 3.5), API (Group I: 0.4 ± 0.18 vs. Group II: 0.37 ± 0.18), SBI (Group I: 0.67 ± 0.15 vs. Group II: 0.65 ± 0.15), the presence of subgingival plaque and bleeding, or the quantity of analyzed bacteria in the subgingival dental plaque samples obtained from the upper left first molars (*p* > 0.05). The oral hygiene status and microbial findings in the upper left first molars in both study groups at all TPs are shown in Supplementary Table S2.

### Effect of Cleaning with Dental Nanoflosses on Oral Hygiene Status

**Fig. 3:**
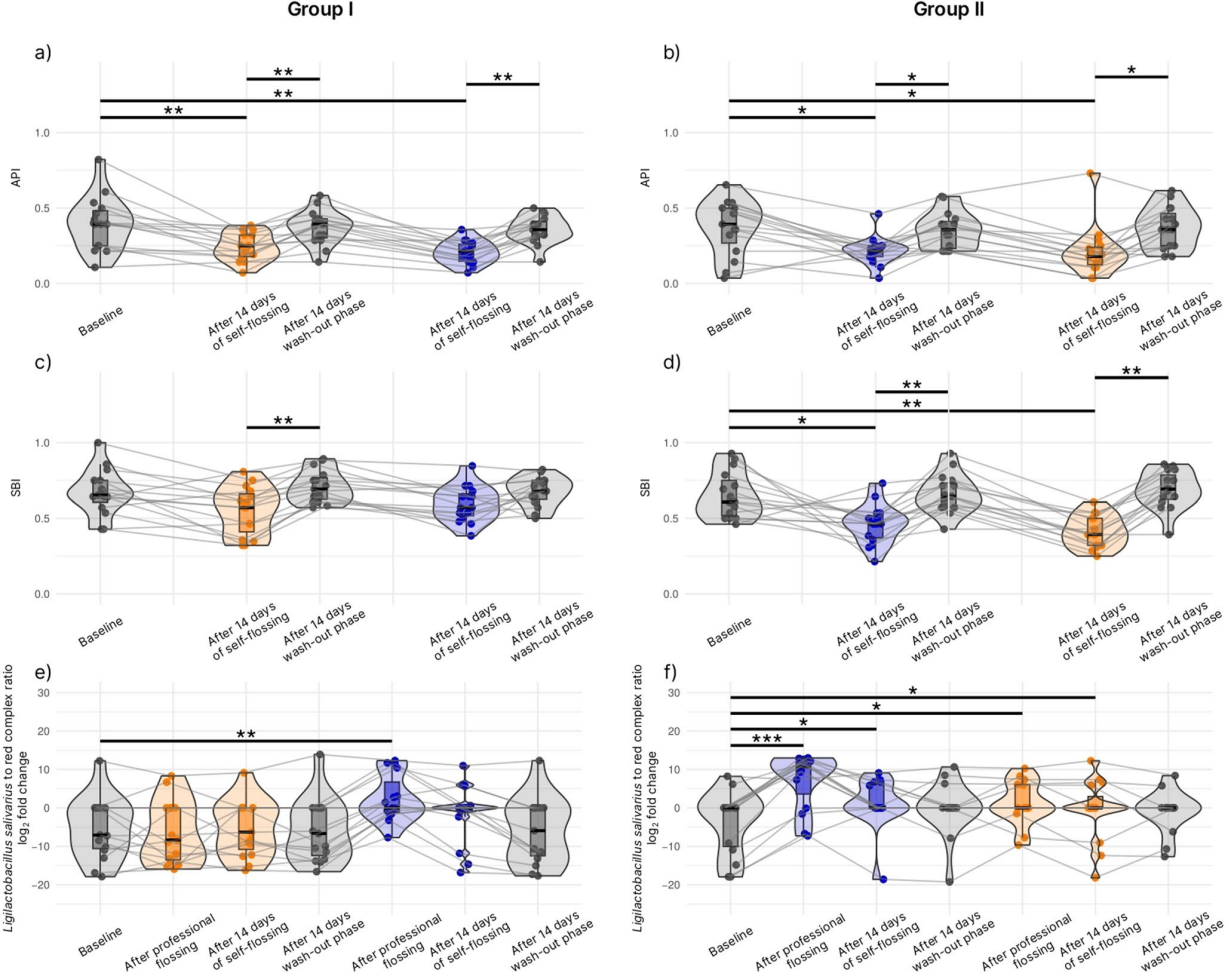
Violin plots with embedded boxplots showing: (**a**, **b**) API, (**c**, **d**) SBI, (**e**, **f**) ratio of concentrations of *Ligilactobacillus salivarius* to red complex bacteria. Orange color indicates periods when participants used the control nanofloss; blue color indicates periods with *LS*-nanofloss. API and SBI were not measured immediately after professional cleaning In each boxplot, the central line indicates the median, the box spans the interquartile range (IQR), and whiskers extend to 1.5 × IQR or to the minimum/maximum value. Statistical comparisons were conducted using paired Wilcoxon signed-rank tests with Benjamini–Hochberg correction for multiple testing. Asterisks indicate significance thresholds: *p* < 0.05 (*), *p* < 0.01 (**), and *p* < 0.001 (***). API, Approximal Plaque Index; SBI, Sulcus Bleeding Index; control nanofloss, probiotic-free nanofloss; *LS*-nanofloss, nanofloss with *Ligilactobacillus salivarius*

In both study groups, the use of dental nanofloss led to a clear improvement in API and SBI, see Fig. 3 and Supplementary Table S2. Although the figures provide an overview of the observed data distribution, all statistical inferences were based solely on mixed-effects models.

**Fig. 4:**
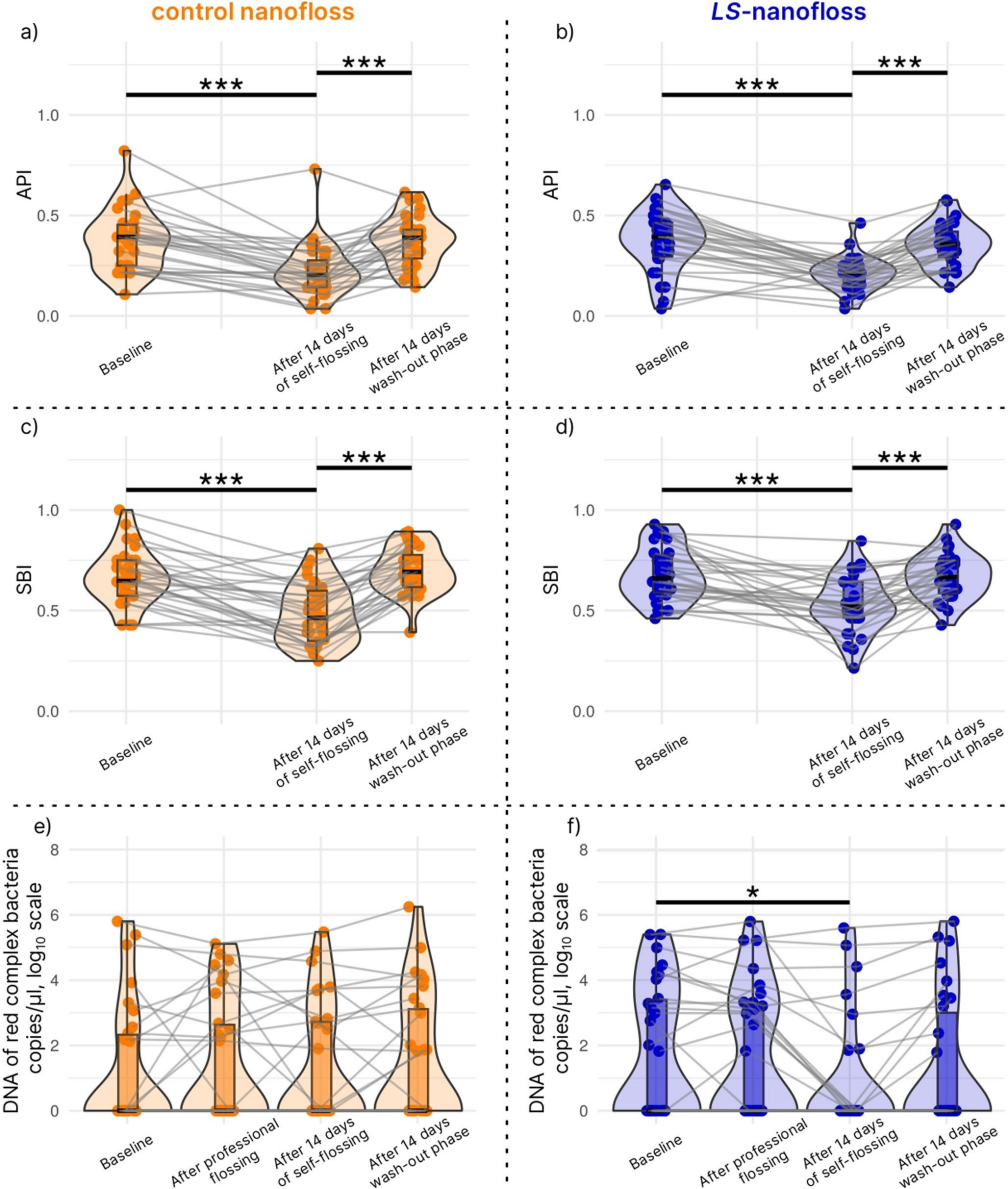
Violin plots with embedded boxplots showing the distributions of (**a**, **b**) API, (**c**, **d**) SBI, and (**e**, **f**) red complex bacterial DNA concentrations in subgingival samples from all participants (*N* = 30) during the use of control nanofloss (left panels) and the *LS*-nanofloss (right panels). API and SBI were not assessed immediately after professional periodontal cleaning In each boxplot, the central line indicates the median, the box spans the interquartile range (IQR), and whiskers extend to 1.5 × IQR or to the minimum/maximum value, whichever is closer. Comparisons between time points were performed using paired Wilcoxon signed-rank tests with Benjamini–Hochberg correction for multiple testing. Asterisks indicate statistical significance thresholds: *p* < 0.05 (*), *p* < 0.01 (**), and *p* < 0.001 (***). API, Approximal Plaque Index; SBI, Sulcus Bleeding Index; control nanofloss, probiotic-free nanofloss; *LS*-nanofloss, nanofloss with *Ligilactobacillus salivarius*

A significant reduction in both clinical indices was observed after the 14-day self-cleaning period, irrespective of the nanofloss used. According to the GLMM analysis (see full results in Table 1), the odds of plaque presence decreased by approximately 56% (API: OR = 0.44, *p* < 0.001), and the odds of gingival bleeding decreased by approximately 57% (SBI: OR = 0.43, *p* < 0.001) relative to baseline. However, these improvements were not maintained during the subsequent wash-out phase, and both indices returned to baseline levels after 14 days without flossing (Figs. 3 and 4). Improvements were markedly site-dependent: anterior teeth showed substantially lower odds of plaque and bleeding compared with posterior teeth (API: OR = 0.34, *p* < 0.001; SBI: OR = 0.40, *p* < 0.001), which is consistent with known anatomical patterns of biofilm accumulation (Supplementary Figure S3, Table 1).

**Table 1:** Outcomes of the linear mixed models for individual studied characteristics, namely treatment (*LS*-nanofloss vs control nanofloss), period (period II vs period I), group (Group II vs Group I), time point within the period (after 14 days home flossing vs before professional flossing), and dental region (anterior vs. posterior teeth). The second option in the brackets always denotes the baseline value. *LS*-nanofloss, nanofloss with *Ligilactobacillus salivarius*; API, Approximal Plaque Index; SBI, Sulcus Bleeding Index; GLMM, Generalized Linear Mixed Model; CI, confidence interval

Observed characteristic	Predictor	estimate	95% CI	*p*-value
*L. salivarius* to red complex bacteria ratio (log2-ratio change)	Intercept	−0.04	[−1.54; 1.45]	0.954
	Treatment	2.16	[0.65; 3.67]	0.007
	Period	−0.55	[−2.06; 0.96]	0.480
	Group	0.29	[−1.22; 1.80]	0.711
*L. salivarius* (log2 relative quantity)	Intercept	−0.49	[−1.15; 0.17]	0.153
	Treatment	1.14	[0.23; 1.72]	0.001
	Period	0.37	[−0.21; 0.94]	0.218
	Group	0.97	[0.23; 1.71]	0.016
Red complex (log2 relative quantity)	Intercept	−0.44	[−1.83; 0.94]	0.529
	Treatment	−1.07	[−2.46; 0.33]	0.141
	Period	0.96	[−0.43; 2.36]	0.182
	Group	0.73	[−0.67; 2.13]	0.310
API (GLMM, odds ratios)	Intercept	0.96	[0.74; 1.23]	0.749
	Treatment	0.96	[0.78; 1.19]	0.739
	Time point (within Period)	0.44	[0.35; 0.56]	< 0.001
	Period	0.89	[0.76; 1.04]	0.152
	Dental region	0.34	[0.29; 0.40]	< 0.001
	Treatment × Time within Period Interaction	0.94	[0.68; 1.30]	0.706
SBI (GLMM, odds ratios)	Intercept	3.09	[2.43; 3.93]	< 0.001
	Treatment	1.08	[0.87; 1.33]	0.500
	Time point (within Period)	0.43	[0.35; 0.53]	< 0.001
	Period	1.06	[0.92; 1.23]	0.429
	Dental region	0.40	[0.34; 0.46]	< 0.001
	Treatment × Time within Period Interaction	1.13	[0.85; 1.53]	0.393

Importantly, the *LS*-nanofloss did not provide additional benefit beyond that of the control nanofloss in these indices, the treatment × time interaction was non-significant for both API (OR = 0.94, *p* = 0.707) and SBI (OR = 1.13, *p* = 0.393). No significant period effects were detected, indicating an absence of carry-over effect.

### Effect of Dental Nanoflosses on Subgingival Bacterial Composition

*L. salivarius* was detected in 93.3% of samples immediately following professional periodontal cleaning, with concentrations ranging from 300 to 10,000 copies/µL. This prevalence declined to 36.7% after two weeks of self-cleaning and further to 13.3% following the wash-out phase. Interestingly, in several participants from Group II, *L. salivarius* DNA reappeared after subsequent cleaning with the control nanofloss. Mixed-effects modeling confirmed that the *LS*-nanofloss produced a significantly greater post-treatment increase in relative quantity of *L. salivarius* than the control floss (CI = [0.23, 1.72], *p* = < 10^− 3^), consistent with the descriptive trends shown in Figs. 3e, f and Supplementary Figure S4. Full results of the mixed-effect models are presented in Table 1.

The relative concentration of red complex bacterial DNA declined after professional cleaning and after self-cleaning; however, the mixed-effects model detected no significant difference between floss types in this change (CI = [−2.46, 0.33], *p* = 0.141), indicating that reductions in red complex bacteria were comparable in both TPs (Figs. 4e, f).

In line with the above, the ratio of *L. salivarius* to red complex bacteria increased significantly more following the use of *LS*-nanofloss than after control flossing (CI = [0.65, 3.67], *p* = 0.007), demonstrating a treatment-related shift toward higher relative quantity of *L. salivarius*. Although a carry-over effect was observed for the red complex bacteria and the *L. salivarius*/red complex bacteria ratio (*p* = 0.029 and *p* = 0.041), demonstrating a difference in the baselines between groups after the first wash-out phase, this does not affect the validity of the within-patient analyses showing superior results for the *LS*-nanofloss over the control floss. Detailed temporal patterns are provided in Supplementary Table S2 and visualized in Supplementary Figure S4.

*S. mutans* was found only in eight samples on the baseline, no statistical analyses on its quantity or presence were performed.

### Correlation of Clinical Parameters with the Presence of Selected Bacteria in the Subgingival Environment

**Fig. 5:**
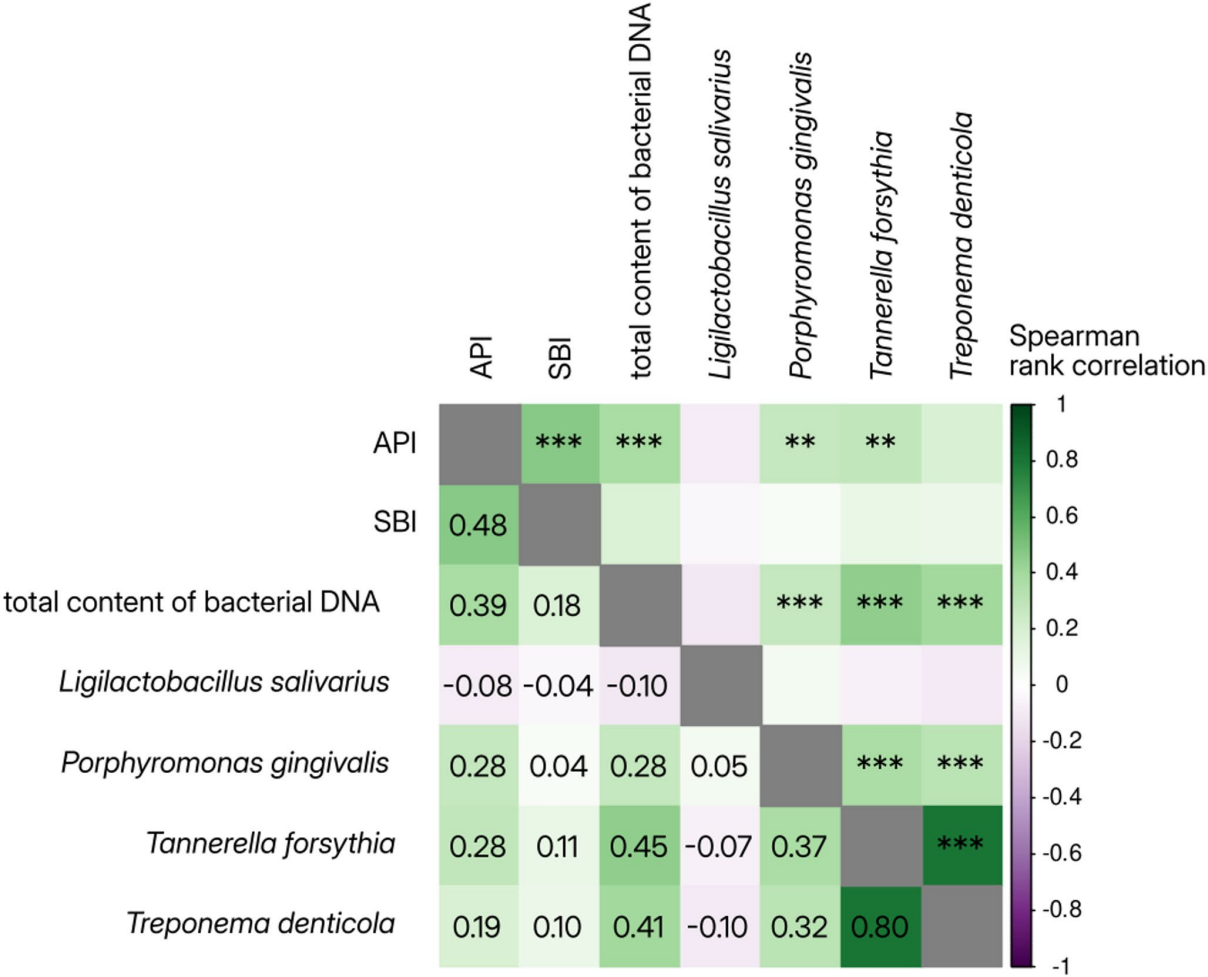
Correlation matrix (Spearman’s rank correlations) showing associations among API, SBI, and DNA concentrations of *Ligilactobacillus salivarius*, *Porphyromonas gingivalis*, *Tannerella forsythia, Treponema denticola*, and the total bacterial content calculated across all patients and time points. Asterisks indicate statistical significance thresholds: *p* < 0.05 (*),* p* < 0.01 (**), and *p* < 0.001 (***). API, Approximal Plaque Index; SBI, Sulcus Bleeding Index

A moderate positive correlation was observed between API and SBI (*r* = 0.48, *p* < 0.001). API also significantly positively correlated with two bacteria of the red complex: *P. gingivalis* and *T. forsythia* (both *r* = 0.28; *p* < 0.001). All three red complex bacteria significantly intercorrelated, with the strongest association observed between *T. denticola* and *T. forsythia* (*r* = 0.80). The full correlation matrix is visualized in Fig. 5.

## Discussion

Dental floss is considered, along with interdental brushes, an essential device for dental hygiene, since sufficient removal of dental plaque from interdental spaces is impossible with manual brush only. Since most dental and periodontal pathologies originate in interproximal areas, interdental cleaning is indispensable in both prevention and treatment of gingivitis, periodontitis, and dental caries [38]. Textured or expanding filaments, such as nanofiber-based materials, have the potential to be more plaque-retentive, and, therefore, more effective than their standards counterparts. Moreover, the nanofiber-coated floss can be also used as a carrier for additional agents, including, as in our case, probiotic bacteria.

In this study, we proved that the introduced nanofiber floss with plaque-retentive probiotic-containing nanosurface is very effective in both subgingival plaque removal and gingival bleeding control. The effect of both types of nanofloss on these parameters was comparable. Still, the effect was short-lived, and cessation of flossing led to fast re-establishment of pre-treatment values of both plaque and bleeding indices. Besides, the effect was more pronounced in the anterior region of dentition than in the posterior area. This was, however, not surprising as this effect is well-known and associated with the accessibility to cleaning of the individual parts [39].

Investigation of the retention of probiotic bacteria and of changes in the presence of red complex bacteria following the use of the nanofloss constituted the principal aims of this study. The fact that immediately after professional flossing with *LS*-nanofloss, DNA of the probiotic strain was present within the gingival sulcus in 93% of subjects, indicates that this is an easy, safe, and user-friendly way to introduce and recolonize periodontal pockets or gingival sulci with the used beneficial bacterial strain. This can be potentially further supported by daily home flossing. After two weeks of patients’ self-care with the probiotic floss, retention of the probiotic strain was observed in 37% of subjects. We assume that deposition of the probiotic from the nanofloss into the sulcus is dependent on proper flossing technique, which might not be adequate in many cases.

Importantly, prolonged retention of the probiotic strain within the sulcus even after 14 days of not using the *LS-*nanofloss was seen in 13% of subjects. This proves that the DNA delivered by flossing with the probiotic-enriched nanofloss belonged at least partially to viable *L. salivarius* bacteria from the nanofloss that were able to integrate with the eubiotic biofilm in the long term. On the other hand, this effect was observed in a relatively low percentage of patients. This is, in our opinion, caused predominantly by the fact that we have intentionally selected a tooth from the posterior part of the dentition (tooth 26) for this analysis, which might be more difficult to floss properly. From this perspective, we consider the tooth 26 to represent the worst-case scenario and even this relatively low number of patients who retained *L. salivarius* even after the wash-out period to be rather a success; it could be expected that in the anterior part, the retention would be even higher.

Several studies have proved positive effects of probiotic replacement therapy on periodontal parameters, which suggests this strategy to be a promising tool in both prevention and therapy of periodontal and dental diseases [20, 22]. In these studies, a single bacterium or a mixture of multiple strains were repeatedly applied subgingivally by the clinician using a blunt syringe [21, 23], chitosan polymers, or microgranule capsules serving as carriers of probiotics [40]. For routine targeted clinical application, however, a more user-friendly device for repeated administration of probiotic bacteria is needed. For non-targeted daily homecare, probiotic-containing lozenges or mouthwashes have been suggested [8, 9, 12]. However, such formulations were shown to have limited access to target subgingival areas, resulting in only minor retention of probiotic cultures and inconclusive clinical effect [8, 10, 11, 41] often not clinically detectable in the long-term, as discussed in recent literature reviews [8, 12]. This also explains why adjunctive use of probiotics to non-surgical periodontal therapy is generally rather not recommended [42].

In our study, suppression of the red complex periopathogens was most evident in samples collected after 14 days of self-flossing with the *LS*-nanofloss, which was not observed after the use of the control nanofloss. This reduction in the relative quantity of these periopathogens was, however, not observed immediately after professional flossing. This is an important point as this demonstrates that this decline in red complex bacteria observed after self-flossing was not caused by a simple mathematical artefact when the introduction of new bacterial DNA necessarily leads to the reduction in the relative quantity of DNA from other bacteria. If this artefact played a major role, we would have observed it particularly strongly after professional flossing with the *LS*-nanofloss (after which *L. salivarius* was introduced in 93% of patients). We have, however, found the decline only after a prolonged period of self-flossing with *LS*-nanofloss, indicating that the repeated introduction of *L. salivarius* indeed played a role in the observed ecological shift in the subgingival environment.

After the wash-out phase following the *LS*-nanofloss use, however, the presence of red complex periopathogens returned to its baseline values. It is possible that 14 days of flossing with the *LS*-nanofloss was not sufficient for establishing a stable probiotic-enriched subgingival environment, which would require a prolonged period of flossing. At least, prolonged self-flossing would presumably delay re-emergence of red complex bacteria as shown in previous studies demonstrating that repeated application resulted in delayed re-emergence of periopathogens, possibly favoring periodontal healing after subgingival debridement [8, 23, 40]. From this perspective, the relatively short-term investigation of the effect of both types of nanofloss might be considered a limitation. On the other hand, given the crossover design and wash-out periods, the duration of the study was 70 days as it was, and its further prolonging would excessively burden the participants, which could lead to increased drop-out.

The fact that self-flossing was dependent on the compliance of patients with the prescribed flossing technique can also be considered a limitation of this study. Indeed, the effects of probiotic administration might be even more significant if performed repeatedly by a dental professional. However, our investigation focused on the real-world applicability of both types of nanofloss and from this perspective, everyday professional cleaning is not feasible. We have, therefore, opted for the worst-case scenario (tooth from the posterior region and self-flossing, despite the expected suboptimal cleaning in some patients). No control group flossing with regular polyamide floss was included in this study, which might also be considered a limitation, as such a group could be useful in evaluating the positive effects of nanocoating itself. However, this was not the aim of the present study, in which we focused on evaluating the effect of the probiotic enrichment of a dental nanofloss on the subgingival environment.

Retrospective registration of the trial can also be considered a limitation; however, the fact that the registration included the original protocol approved by the Ethics Committee prior to the study initiation ensures that no post-hoc analytical or clinical decisions were made, ensuring the scientific integrity and transparency.

The main strengths of this trial lie in its robust protocol with a crossover double-blind design and a highly homogeneous study population selected based on strict criteria. Furthermore, our study aimed to investigate the possible use of *LS*-nanofloss in the prevention of periodontitis, not its treatment, which is why all participants were periodontally healthy. Although individuals with mild periodontitis were allowed by the inclusion criteria, only periodontally healthy individuals were in the end included in the study as all individuals with periodontitis considered for inclusion failed to meet all other criteria (mostly due to the use of other medications in patients with periodontitis). However, based on the results of this study, we plan to also perform a study focusing on the adjunctive use of *LS*-nanofloss during periodontitis treatment in the future. Another possible direction of future research might focus on the use of the developed nanofloss for targeted administration of many other bioactive agents (such as antimicrobial peptides or regenerative biomaterials) into the gingival sulcus, which might amplify their local effect. Providing confirmation of our results in larger trials, probiotic-containing nanofloss can become a valid and clinically meaningful tool.

## Conclusions

The probiotic-containing nanofloss was found to have the potential to effectively deliver viable probiotic bacteria subgingivally in an easy, user-friendly, and targeted way, and reduce the amount of red complex periopathogens. Although the effects largely reverted to the original condition after the 14-day wash-out phase, the probiotic-containing nanofiber-based dental floss proved to be a potentially promising instrument for ecological modulation in the subgingival environment (i.e., guided periodontal pocket recolonization) that can be used in both prevention and treatment of periodontitis.

## Supplementary Information

Below is the link to the electronic supplementary material.

ESM 1 **Supplementary Figure S1. **Flowchart of participant enrollment. Control nanofloss, probiotic-free nanofloss; *LS*-nanofloss, nanofloss with *Ligilactobacillus salivarius*. (PDF 505 KB)

ESM 2 **Supplementary Figure S2.** Violin plots with embedded boxplots showing (a,b) *Ligilactobacillus salivarius* DNA concentration and (c,d) red complex bacterial DNA concentration across all study time points in Group I (*N* = 15) and Group II (*N* = 15). Orange color indicates periods when participants used the control nanofloss; blue color indicates periods with *LS*-nanofloss. In each boxplot, the central line indicates the median, the box spans the interquartile range (IQR), and whiskers extend to 1.5 × IQR or to the minimum/maximum value. Statistical comparisons were conducted using paired Wilcoxon signed-rank tests with Benjamini–Hochberg correction for multiple testing. Asterisks indicate significance thresholds: *p* < 0.05 (*), p < 0.01 (**), and *p *< 0.001 (***). Control nanofloss, probiotic-free nanofloss; *LS*-nanofloss, nanofloss with *Ligilactobacillus salivarius*. (PDF 816 KB)

ESM 3 **Supplementary Figure S3. **Changes in the presence of subgingival plaque (a-d) and bleeding (e-g) across all teeth at four time points. Orange frames indicate periods of the control nanofloss usage, blue frames the periods of flossing with the *LS*-nanofloss, the panels without frames indicate the wash-out phases. Each bar (horizontal axes) represents an individual tooth position. The green color indicates the proportion of participants in whom the condition disappeared (i.e., condition improvement), grey indicates no change, and red indicates the proportion of participants in whom the condition appeared (i.e., deterioration) during the particular period. Statistical significance of the observed changes in each period was assessed using binomial tests. Control nanofloss, probiotic-free nanofloss; *LS*-nanofloss, nanofloss with *Ligilactobacillus salivarius*. (PDF 535 KB)

ESM 4 **Supplementary Figure S4. **Heatmap of relative quantification, measured by qPCR, of *Ligilactobacillus salivarius*, *Streptococcus mutans*, *Porphyromonas gingivalis*, *Tannerella forsythia*, and *Treponema denticola* (collectively referred to as the red complex bacteria) in subgingival samples collected from the upper left first molar. The presence of subgingival plaque and subgingival bleeding at the sampled site is also indicated. Rows are grouped by study group, and columns correspond to different time points throughout the study. Orange frames indicate periods of the control nanofloss usage, blue frames the periods of flossing with the *LS*-nanofloss, the panels without frames indicate the wash-out phases. Control nanofloss, probiotic-free nanofloss; *LS*-nanofloss, nanofloss with *Ligilactobacillus salivarius*. (PDF 39.4 KB)

ESM 5 **Supplementary Table S1. **Parameters for the multiplex qPCR panel [33], which was modified in this study by adding *Ligilactobacillus salivarius*. (DOCX 13.3 KB)

ESM 6 **Supplementary Table S2. **Descriptive statistics of key variables in Group I and Group II across all time points in the study. Baseline differences between groups were assessed using the Wilcoxon rank-sum test for continuous variables and the test of proportions for binary variables. *P*-values were adjusted for multiple testing using the Benjamini–Hochberg method. Control nanofloss, probiotic-free nanofloss; *LS*-nanofloss, nanofloss with *Ligilactobacillus salivarius*; ^#^, performed only for upper left first molars; SD, standard deviation; five number summary, [minimum; 1st quartile; median; 3rd quartile; maximum]; NA, non-applicable.(XLSX 15.6 KB)

ESM 7 **Supplementary Table S3.** Raw data. (XLSX 39.0 KB)

## Data Availability

Raw qPCR data (cycle threshold values and concentrations in copies/µL) for each patient and timepoint are provided in the Supplementary Information (Supplementary Table S3).

## Declarations

### Study Registration

This clinical trial was retrospectively registered at ClinicalTrials.gov with ID NCT07149493 on 02/09/2025.

### Retrospective Registration Note

This study was originally designed as a method validation investigation to refine subgingival probiotic delivery techniques using a newly developed nanofiber dental floss containing *Ligilactobacillus salivarius*. At the time of ethics approval and initiation (February 2022), the study was not classified as a clinical trial. However, subsequent interpretation of NIH guidelines suggests that the randomized, blinded, crossover design involving a health-related intervention meets the criteria for interventional clinical research. Accordingly, the investigators now recognize that the study should be registered as a clinical trial. The research team is submitting this retrospective registration to ensure transparency and support future publication. No participant-level outcomes have been modified post hoc, and all data were collected prospectively under full ethical oversight.
