# Supplementary figures and images for: Probiotic-Containing Nanofiber-Based Dental Floss Suppresses Subgingival Red Complex Periopathogens: A Randomized Double-Blind Crossover Trial
Source: Probiotics Antimicrob Proteins. 2026 Jan 4;18(5):6662–75. doi: 10.1007/s12602-025-10898-4 (PMC13369690; doi:10.1007/s12602-025-10898-4)

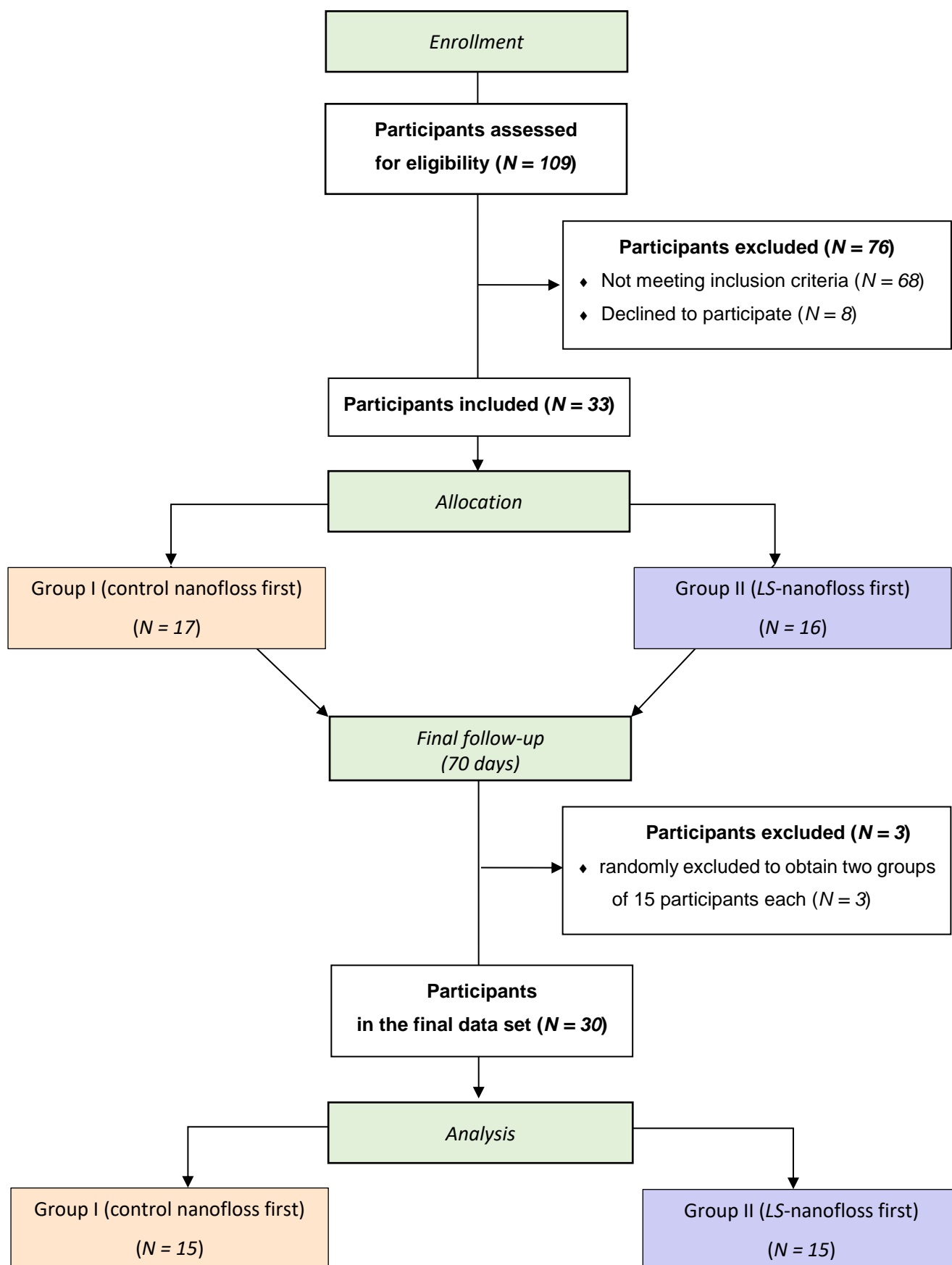

Supplement: Supplementary file 1 — Supplementary Figure S1. Flowchart of participant enrollment. Control nanofloss, probiotic-free nanofloss; LS-nanofloss, nanofloss with Ligilactobacillus salivarius. (PDF 505 KB) [file 12602_2025_10898_MOESM1_ESM.pdf]

Group I

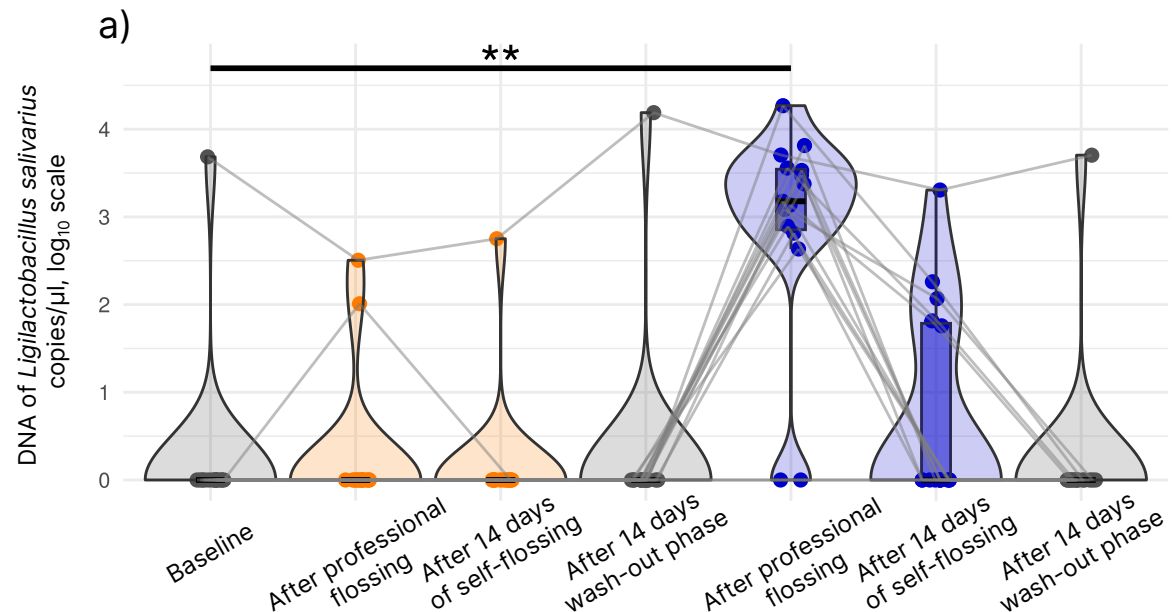

Group II

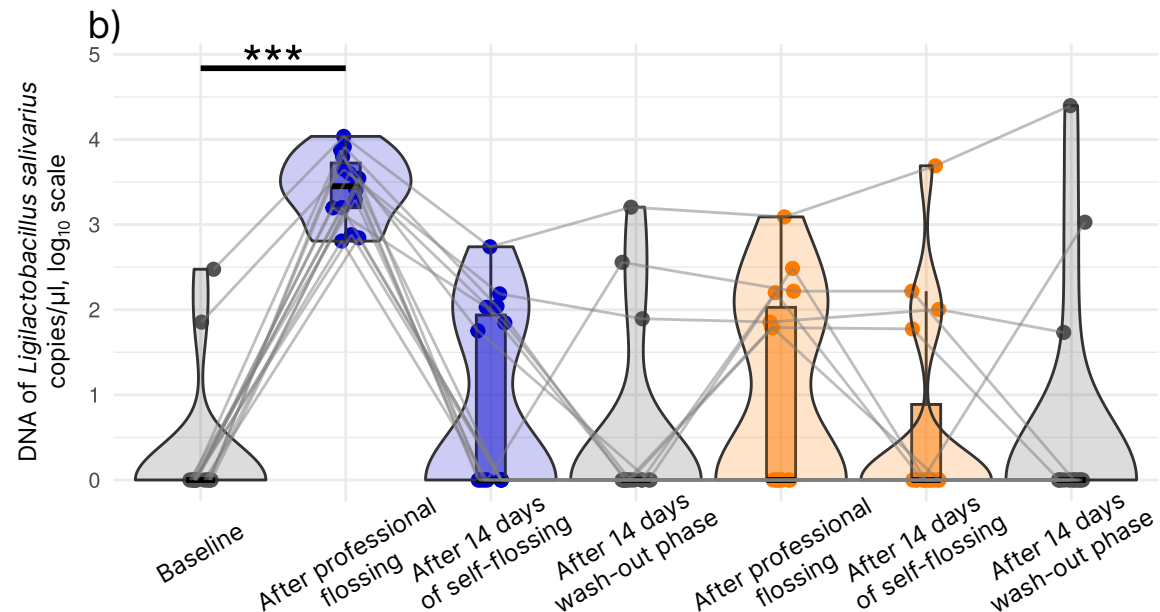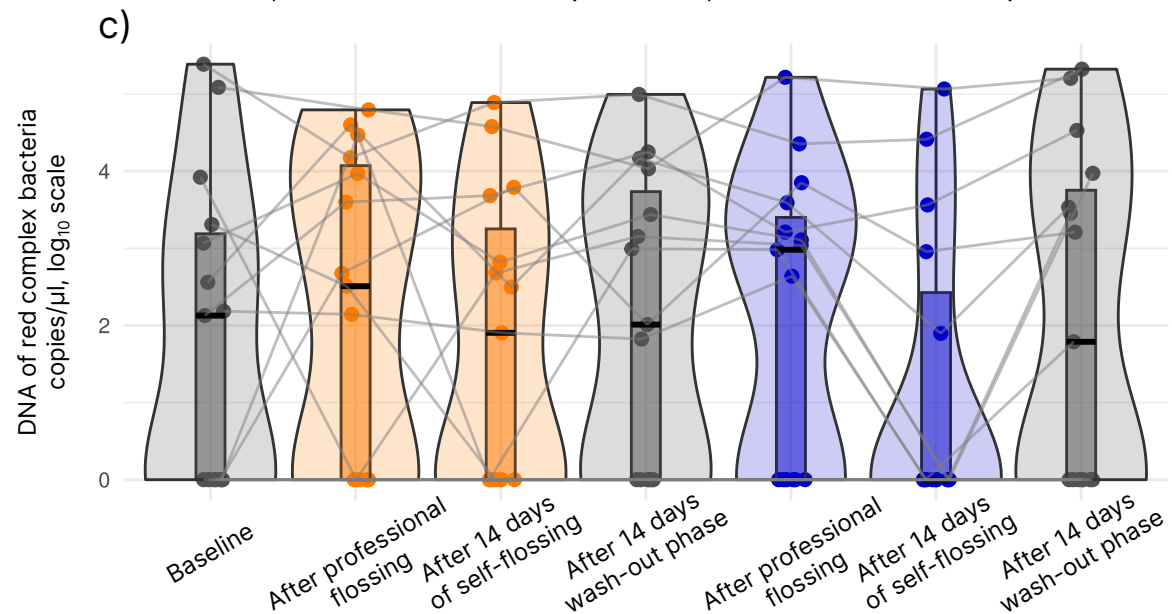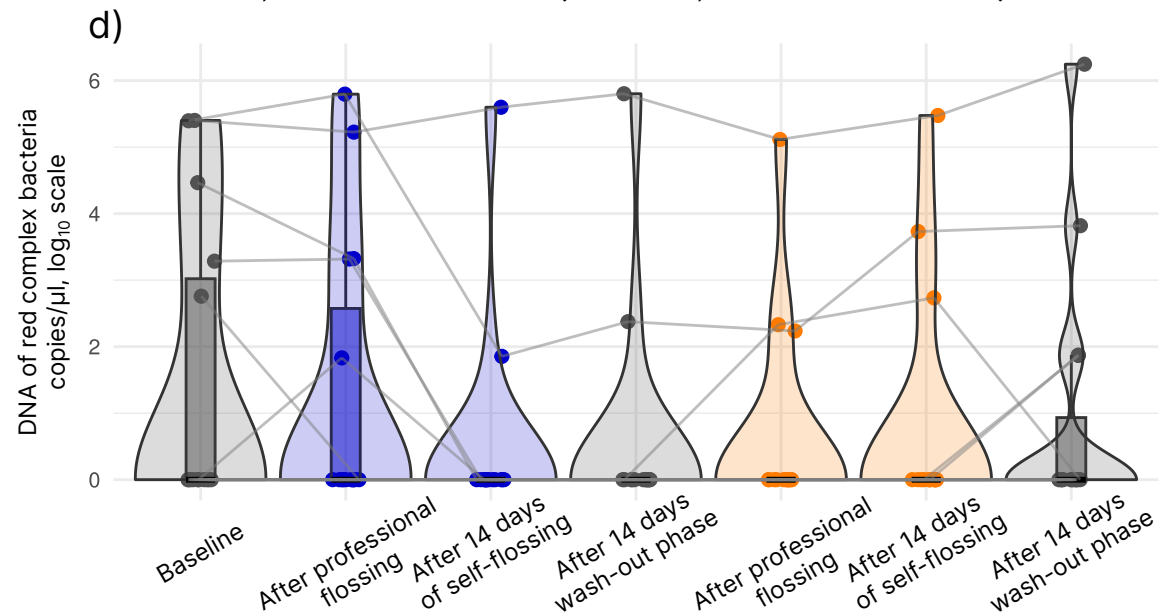

Supplement: Supplementary file 2 — Supplementary Figure S2. Violin plots with embedded boxplots showing (a,b) Ligilactobacillus salivarius DNA concentration and (c,d) red complex bacterial DNA concentration across all study time points in Group I (N = 15) and Group II (N = 15). Orange color indicates periods when participants used the control nanofloss; blue color indicates periods with LS-nanofloss. In each boxplot, the central line indicates the median, the box spans the interquartile range (IQR), and whiskers extend to 1.5 × IQR or to the minimum/maximum value. Statistical comparisons were conducted using paired Wilcoxon signed-rank tests with Benjamini–Hochberg correction for multiple testing. Asterisks indicate significance thresholds: p < 0.05 (*), p < 0.01 (**), and p < 0.001 (***). Control nanofloss, probiotic-free nanofloss; LS-nanofloss, nanofloss with Ligilactobacillus salivarius. (PDF 816 KB) [file 12602_2025_10898_MOESM2_ESM.pdf]

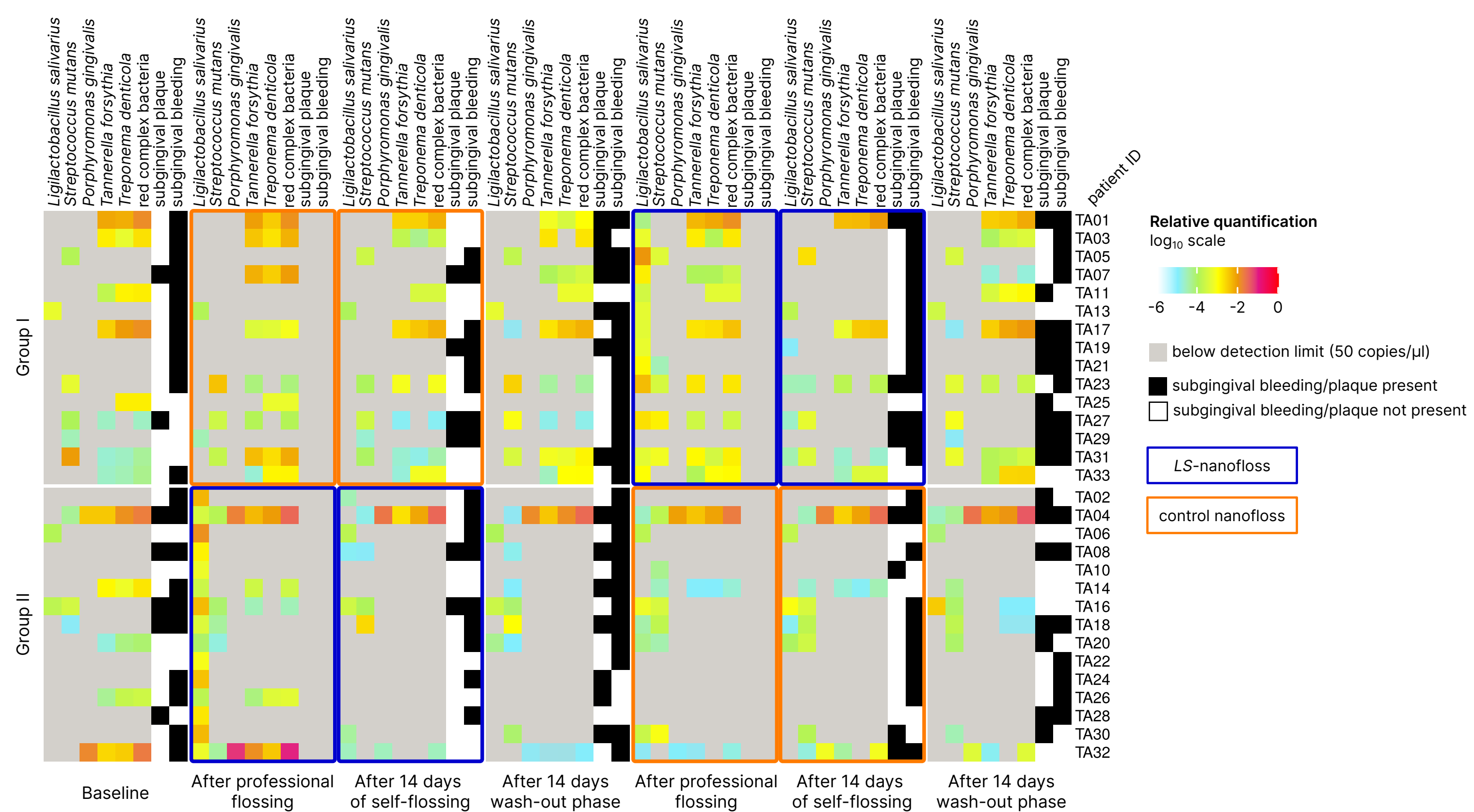

Supplement: Supplementary file 4 — Supplementary Figure S4. Heatmap of relative quantification, measured by qPCR, of Ligilactobacillus salivarius, Streptococcus mutans, Porphyromonas gingivalis, Tannerella forsythia, and Treponema denticola (collectively referred to as the red complex bacteria) in subgingival samples collected from the upper left first molar. The presence of subgingival plaque and subgingival bleeding at the sampled site is also indicated. Rows are grouped by study group, and columns correspond to different time points throughout the study. Orange frames indicate periods of the control nanofloss usage, blue frames the periods of flossing with the LS-nanofloss, the panels without frames indicate the wash-out phases. Control nanofloss, probiotic-free nanofloss; LS-nanofloss, nanofloss with Ligilactobacillus salivarius. (PDF 39.4 KB) [file 12602_2025_10898_MOESM4_ESM.pdf]
