# Supplementary material for: Probiotic-Containing Nanofiber-Based Dental Floss Suppresses Subgingival Red Complex Periopathogens: A Randomized Double-Blind Crossover Trial
Source: Probiotics Antimicrob Proteins. 2026 Jan 4;18(5):6662–75. doi: 10.1007/s12602-025-10898-4 (PMC13369690; doi:10.1007/s12602-025-10898-4)
Supplement: Supplementary file 3 — Supplementary Figure S3. Changes in the presence of subgingival plaque (a-d) and bleeding (e-g) across all teeth at four time points. Orange frames indicate periods of the control nanofloss usage, blue frames the periods of flossing with the LS-nanofloss, the panels without frames indicate the wash-out phases. Each bar (horizontal axes) represents an individual tooth position. The green color indicates the proportion of participants in whom the condition disappeared (i.e., condition improvement), grey indicates no change, and red indicates the proportion of participants in whom the condition appeared (i.e., deterioration) during the particular period. Statistical significance of the observed changes in each period was assessed using binomial tests. Control nanofloss, probiotic-free nanofloss; LS-nanofloss, nanofloss with Ligilactobacillus salivarius. (PDF 535 KB) [file 12602_2025_10898_MOESM3_ESM.pdf]

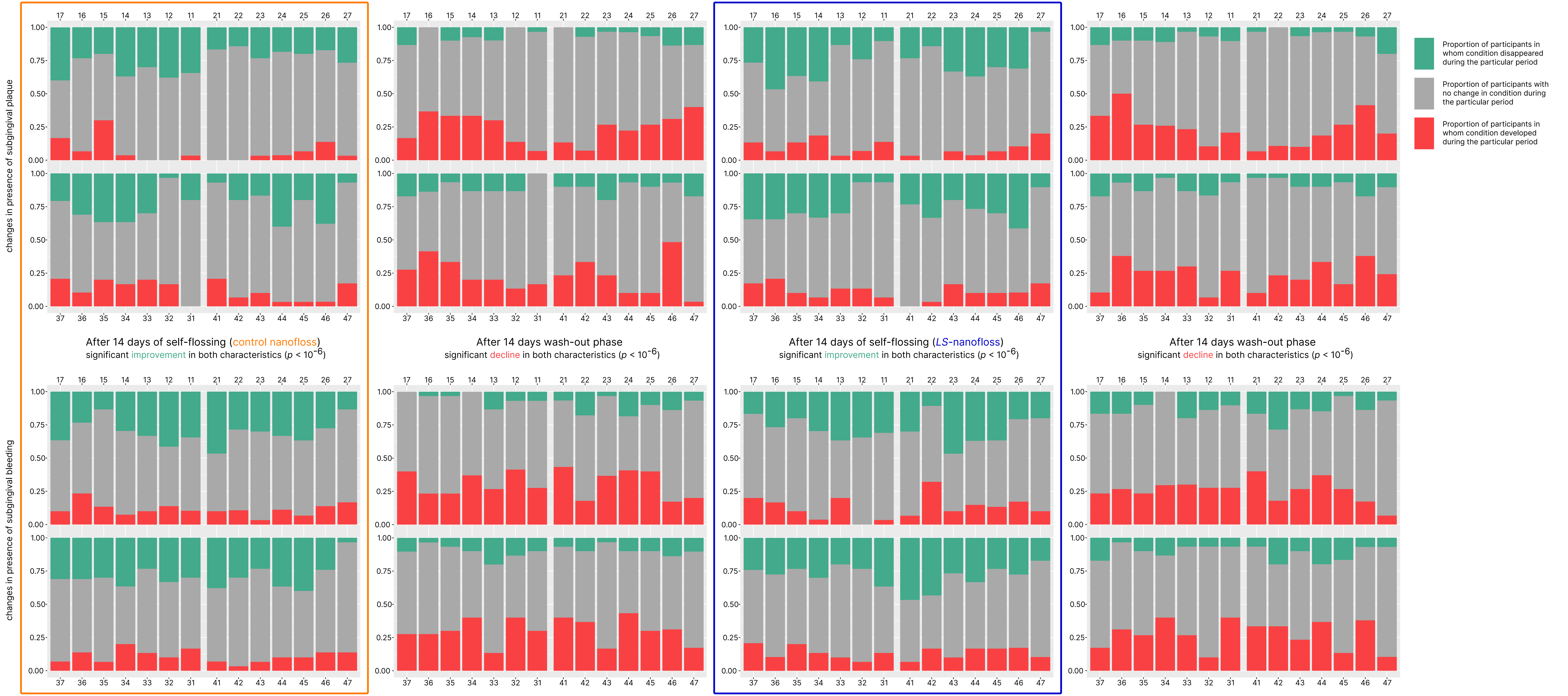

changes in presence of subgingival plaque
changes in presence of subgingival bleeding
After 14 days of self-flossing (control nanofloss)
significant improvement in both characteristics ($p < 10^{-6}$)
After 14 days wash-out phase
significant decline in both characteristics ($p < 10^{-6}$)
After 14 days of self-flossing (*LS*-nanofloss)
significant improvement in both characteristics ($p < 10^{-6}$)
After 14 days wash-out phase
significant decline in both characteristics ($p < 10^{-6}$)
Proportion of participants in whom condition disappeared during the particular period
Proportion of participants with no change in condition during the particular period
Proportion of participants in whom condition developed during the particular period
17 16 15 14 13 12 11 21 22 23 24 25 26 27
37 36 35 34 33 32 31 41 42 43 44 45 46 47
0.00 0.25 0.50 0.75 1.00
