# Supplementary material for: Probiotic-Containing Nanofiber-Based Dental Floss Suppresses Subgingival Red Complex Periopathogens: A Randomized Double-Blind Crossover Trial
Source: Probiotics Antimicrob Proteins. 2026 Jan 4;18(5):6662–75. doi: 10.1007/s12602-025-10898-4 (PMC13369690; doi:10.1007/s12602-025-10898-4)
Supplement: Supplementary file 5 — Supplementary Table S1. Parameters for the multiplex qPCR panel [33], which was modified in this study by adding Ligilactobacillus salivarius. (DOCX 13.3 KB) [file 12602_2025_10898_MOESM5_ESM.docx]

**Supplementary Table S1.** Parameters for the multiplex qPCR panel [33], which was modified in this study by adding *Ligilactobacillus salivarius*.

| **Bacterial strain** | **Target region/gene** | | **Type of oligonucleotide** | | **Oligonucleotide name** | | **Specific primers and probe** | | **Fluorophore** | | **Amplicon size (bp)** | | **Concentration in reaction** |
| --- | --- | --- | --- | --- | --- | --- | --- | --- | --- | --- | --- | --- | --- |
|  |  | | forward | | LS_16S_23S_F | | TAC ACC GAA TGC TTG CAT TCA | |  | |  | | 0.4 mM |
| *Ligilactobacillus salivarius* | 16S-23S | reverse | | LS_16S_23S_R | | AGG ATC ATG CGA TCC TTA GAG A | |  | | 138 | | 0.25 mM | |
|  |  | | probe | | LS_16S_23S_FAM | | CCGTAAGAAGTTGAGTGGCGG | | FAM | |  | | 0,2 mM |
|  |  | | forward | | SM_gtfB_F | | CCTACAGCTCAGAGATGCTAT | |  | |  | | 0.25 mM |
| *Streptococcus mutans* | *gtfB* | | reverse | | SM_gtfB_R | | GCCATACACCACTCATGAATT | |  | | 113 | | 0.25 mM |
|  |  | | probe | | SM_gtfB_FAM | | TGGAAATGACGGTCGCCGTTAT | | Cy5 | |  | | 0.2 mM |

[33] Lochman J, Zapletalova M, Poskerova H, Izakovicova Holla L, Borilova Linhartova P. Rapid Multiplex Real-Time PCR Method for the Detection and Quantification of Selected Cariogenic and Periodontal Bacteria. Diagnostics (Basel, Switzerland). 2019;10(1). doi: 10.3390/diagnostics10010008.
